# Deep-UV laser direct writing of photoluminescent ZnO submicron patterns: an example of nanoarchitectonics concept

**DOI:** 10.1080/14686996.2022.2116294

**Published:** 2022-10-07

**Authors:** Quentin Kirscher, Samar Hajjar-Garreau, Fabien Grasset, Dominique Berling, Olivier Soppera

**Affiliations:** a Institut de Science des Matériaux de Mulhouse (IS2M) UMR 7361 CNRS-UHA, Université de Haute Alsace, Mulhouse, France; b Université de Strasbourg, Strasbourg, France; c CNRS-Saint Gobain-NIMS, IRL 3629, Laboratory for Innovative Key Materials and Structures (LINK), National Institute for Materials Science (NIMS), Tsukuba, Japan; d Université Rennes, CNRS, ISCR, UMR6226, Rennes, France

**Keywords:** ZnO, nanocrystals, photolithography, photoluminescence, optical coatings, nanoarchitectonics

## Abstract

Micro- and nanopatterning of metal oxide materials is an important process to develop electronic or optoelectronic devices. ZnO is a material of choice for its semiconducting and photoluminescence properties. In the frame of the nanoarchitectonics concept, we have developed and investigated a new process that relies on direct writing laser patterning in the Deep-UV (DUV) range to prepare photoluminescent microstructures of ZnO at room temperature, under air. This process is based on a synthesis of colloidal ZnO nanocrystals (NCs) with a careful choice of the ligands on the surface to obtain an optimal (i) stability of the colloids, (ii) redissolution of the non-insolated parts and (iii) cross-linking of the DUV-insolated parts. The mechanisms of photocrosslinking are studied by different spectroscopic methods. This room temperature process preserves the photoluminescence properties of the NCs and the wavelength used in DUV allows to reach a sub-micrometer resolution, which opens new perspectives for the integration of microstructures on flexible substrates for optoelectronic applications.

## Graphical abstract

**Figure:**
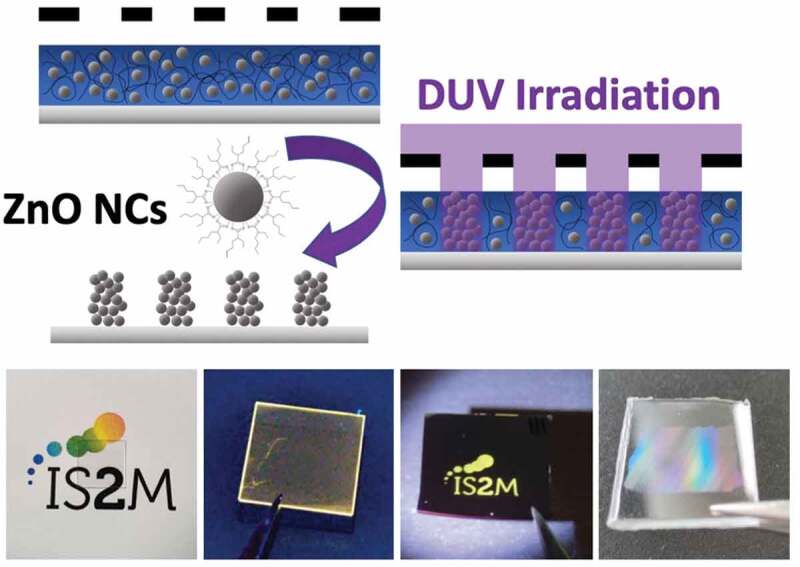

## 1. Introduction

Metal oxide thin films play a very important role in many applications such as protective coatings, electronic devices, optical layers, gas sensors, biosensors, photodetectors, etc. [1,2]. These films are usually fabricated by physical vapor deposition (sputtering, evaporation, laser ablation), chemical vapor deposition (ALD, CVD) or chemical solution deposition (sol-gel). The development of new methods of preparation, simple, respectful of the environment while preserving the optoelectronic properties of thin films are sought. In recent years, the compatibility of these processes with micro- or nanostructuring steps, large surfaces and flexible substrates has become a major scientific and industrial issue.

In the nanoarchitectonic context [3,4], solution chemistry approaches address a large part of these issues in particular, by opening up to metal oxide materials while maintaining so-called soft chemistry processes, *e.g*. with relatively low-temperature treatments. These approaches are usually based on molecular building blocks [3]. Alternatively, colloidal nanocrystals (NCs) have been proposed as building blocks to elaborate thin films while keeping the interests of a soft chemistry approach [5]. Colloidal NCs are indeed a versatile platform for the construction of electronic and optoelectronic devices. These materials have enabled the low-temperature deposition to produce, for example, light-emitting diodes (LEDs), field-effect transistors (FETs), near- and mid-range photodetectors or solar cells, based on noble metallic colloidal NCs [6,7], semiconductors [8–10], or dielectrics [11,12]. In addition to their chemical composition, size and shape, the properties of the materials manufactured on the basis of these NCs also depend on the nature of the interactions between the NCs, which is directly related to the nature of the surface ligands and the associated processing method [13,14]. The use of specific ligands, either organic or inorganic, allows to ensure a continuity of properties by a better coupling between NCs, which has been used to obtain layers with high conductivity or electrical mobility for electrical or optoelectronic applications [15].

Light-sensitive ligands can also be used to trigger the connection between NCs via light and thus realize photoresist based on colloidal solutions of NCs. Y. Wang *et* *al.* presented a chemical approach for direct optical lithography of functional inorganic nanomaterials without photoresist [16]. In this approach, anionic or cationic ligands can be activated under irradiation via photoacid generators (PAGs), allowing for patterning.

More recently, J.-A. Pan *et* *al.* proposed photosensitive oxime sulfonate esters (-C═N-OSOO-) as photosensitive ligands. Under the action of blue light (405 nm), this ligand decomposes at the N-O bond, which causes a decrease in the solubility of NCs or quantum dots, used to prepare patterns [17]. However, this very elegant approach requires the synthesis of specific ligands grafted on the surface of the NCs.

**Figure 1.**
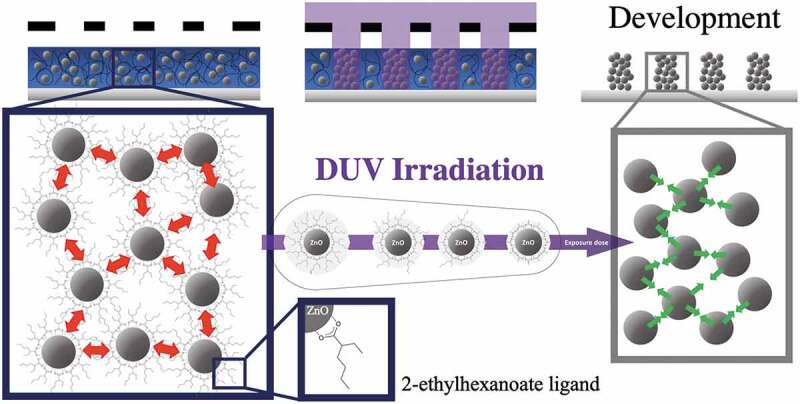
Schematic of DUV photopatterning showing the role of 2-ethylhexanoate ligand in the photolithography process.

In this paper, we propose a new approach based on the use of Deep-Ultraviolet (DUV, λ = 193 nm) photolithography and the use of commercial zinc-based surface modifier ligands. We show that this type of radiation allows to induce a decrease of the solubility of surface-modified NCs deposited as thin films for photolithography applications. This approach is based on a controlled synthesis of colloidal ZnO NC solutions. In particular, a study has been carried out to define the choice of appropriate ligands that allow both the preparation of optical quality thin films by spin-coating, a photosensitivity in the DUV range that makes the film insoluble in the insolated parts while retaining the possibility of redissolving the film in the non-insolated parts (Figure 1).

As illustrated in Figure 1, the objective of this paper is to show that in the irradiated areas, DUVphotolysis of the ligands which modifies the NC interactions: the repulsive interactions arising from the presence of the ligands around the NCs disappear following DUV irradiation, which causes the aggregation of the NCs. The exposed parts become insoluble, which corresponds to a negative tone resin. The interest of this approach also lies in the possibility to easily generate patterns at the sub-micrometer scale. Finally, we demonstrate that the conditions used to perform the photolithography step allow to keep the photoluminescence properties of the ZnO NCs as in solution.

## 2. Experimental section

### 2.1. Materials

Zinc acetate dihydrate (Zn(CH_3_COO)_2_ . 2H_2_O, Sigma Aldrich), tetramethylammonium hydroxide (TMAH, 25 wt.% in methanol, Alfa Aesar), zinc propionate (C_6_H_10_O_4_Zn, Thermo Fischer), zinc 2-ethylhexanoate (C_16_H_30_O_4_Zn, Zn   20%, cont. 1% diethylene glycol monomethyl ether, Alfa Aesar), zinc stearate (C_36_H_70_O_4_Zn, Aldrich), diethyl ether (Et_2_O, Aldrich) and anhydrous 1-propanol (Aldrich) were used as received.

### 2.2. Synthesis of ZnO nanocrystals (NCs)

The protocol is adapted from [18] and schematically presented in Figure S1. About 20 mmol Zn(CH_3_COO)_2_ . 2H_2_O is dispersed in 40 ml of anhydrous propanol. The mixture is heated in an oil bath to 125°C and distilled on a rotary evaporator for about 7 minutes. Nine milliliters of TMAH at 2.375 M is quickly added to the still hot solution, and the mixture is stirred vigorously by hand 1 minute until the complete dissolution of the white precipitate formed upon addition of the TMAH base. The solutions are left under stirring for 48 h to mature the NCs in solution. Then, the NCs are precipitated by adding 40 ml of Et_2_O and the solutions are centrifuged for 15 min at 8000 rpm at 10°C to remove the supernatant containing unreacted precursor species. The yield of the reaction was estimated to be around 36% by ICP-OES chemical analysis. The NCs are finally redispersed in a suitable volume (20 ml) of 1-propanol to adjust the concentration at 0.36 M of ZnO in the colloids. The solution is stirred few minutes until total redispersion of the solid and clear colloids are obtained. The obtained colloidal solution is stored at 4°C and stable for several months.

The synthesis with the three zinc-based surface modifier precursors was carried out using two different procedures. For C_6_H_10_O_4_Zn and C_16_H_30_O_4_Zn, 3.5% and 6.9 mol% were added, respectively, into the colloidal solution at the end of the procedure. For C_36_H_70_O_4_Zn, 6.9% (molar %) were added at the beginning and mixed with Zn(CH_3_COO)_2_ . 2H_2_O, before the distillation.

The solutions were characterized by high-resolution transmission electron microscopy (HRTEM; ARM200F JEOL microscope). A drop of solution diluted 1000 times was deposited on a metal grid covered with a Si_3_N_4_ membrane (Ted Pella).

### 2.3. Thin film deposition

The films are deposited by spin-coating (Süss Microtech Delta6) on different substrates: silicon wafers (Siegert Wafer) cut into pieces of about 2 cm × 2 cm, quartz substrates of 2 cm × 2 cm × 2 mm (Ted Pella), glass slides (VWR International bvba), polycarbonate sheets (Bayer MaterialScience) cut into pieces of about 2.5 cm × 2.5 cm. Depositions are performed under ambient atmospheric conditions (20°C and relative humidity between 20% and 40%). Before deposition, the colloidal solutions are stirred under ultrasound and filtered through a 0.20 µm polytetrafluoroethylene membrane. One hundred microliters of solution are deposited on the cleaned substrate before rotation. The thickness of the film obtained can be adjusted according to the rotation speed (Figure S2).

### 2.4. Laser and thermal annealing

The ZnO NCs thin films are treated by laser irradiation at 193 nm with an Excistar 500 laser (Coherent). The irradiance of the laser beam at the sample surface is maintained at 4.5 ± 0.5 mW.cm^-2^. The dose is controlled by setting the exposure time via an automatically triggered mechanical shutter.

For microstructuring, a proximity printing configuration was adopted using a chrome mask on a fused silica substrate. After exposure, the sample was immersed in a bath of 1-propanol, the same solvent used to disperse the colloids, for 60 seconds under stirring and then dried under a low flow of dry air. In the case of patterns with a period of 500 nm, the structuring is obtained by interference lithography using a phase mask with a period of 1000 nm.

In parallel, heat treatments were conducted by annealing on a hot plate in air for 1 hour. The reference temperature is taken at 300°C, but other temperatures were also tested.

### 2.5. Characterization methods

Measurements of the film thickness were performed by spectroscopic ellipsometry using a UVISEL ellipsometer from Horiba-Jobin-Yvon (spectral range from 190 to 830 nm). Obtained data were fitted using Kato-Adachi model with the DeltaPsi 2 software from the UVISEL ellipsometer.

The Fourier-transform infrared spectroscopy (FTIR) measurements were performed with a Thermo Scientific Nicolet 8700 system. Films were deposited on thinner silicon wafers (thickness around 250 µm or less). Transmission mode was used to collect the data. Spectra were taken with a resolution of 4 cm^−1^ and were averaged over 16 scans in the range 4000–400 cm^−1^.

The X-ray photoelectron spectroscopy (XPS) analysis was performed on a Gammadata Scienta (Uppsala, Sweden) SES 200–2 X-ray photoelectron spectrometer under ultrahigh vacuum conditions (P < 10^−7^ Pa). CASAXPS (Casa Software Ltd, Teignmouth, UK, www.casaxps.com) was used to fit all the peaks and area of each component of XPS data.

The microstructured films were characterized using atomic force microscopy (AFM) in the tapping mode, using a FlexAFM model from Nanosurf with uncoated Si ACT-50 cantilever from AppNano with a spring constant between 13 and 77 N.m^−1^. Scanning was performed at 1 line/s with an image resolution of 256 × 256 pixels.

Grazing incidence X-ray diffraction (GIXRD) patterns of the thin films were measured on a SmartLab diffractometer (Rigaku Corp., Tokyo, Japan) with copper Kα radiation (λ = 1.5406 Å), operated at 50 mA and 40 kV. The angle of incidence (ω) was set between 0.2° and 0.8°.

Absorption spectra were recorded on a Lambda 950 UV/Vis spectrometer from the Perkin Elmer (spectral range 200–800 nm). Thin films were deposited on quartz substrates.

Inductively coupled plasma-optical emission spectrometry (ICP-OES) was carried out using iCAP 7000Series (Thermo Scientific) under argon flux.

Photoluminescence was measured using a picosecond laser diode (Jobin Yvon deltadiode, 375 nm) and a QEPRO spectrometer (Ocean Optics). Excitation vs emission maps were recorded with a Horiba Duetta spectrophotometer.

## 3. Results and discussion

**Figure 2.**
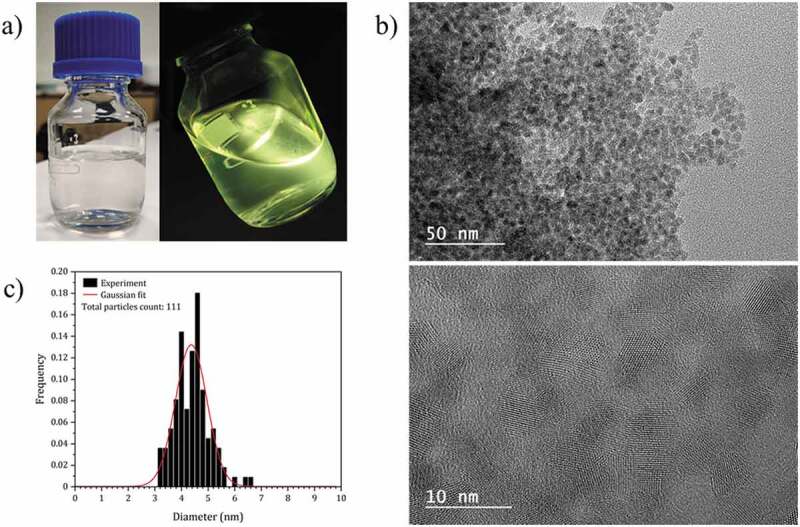
(a) Photographs of the colloidal solution of ZnO NCs capped with 2-ethylhexanoate ligands, under visible (left) and 365 nm UV illumination (right). (b) HRTEM images of the ZnO NCs functionalized with 2-ethylhexanoate ligands. (c) Particle size distribution of the ZnO NCs functionalized with 2-ethylhexanoate ligands.

**Figure 3.**
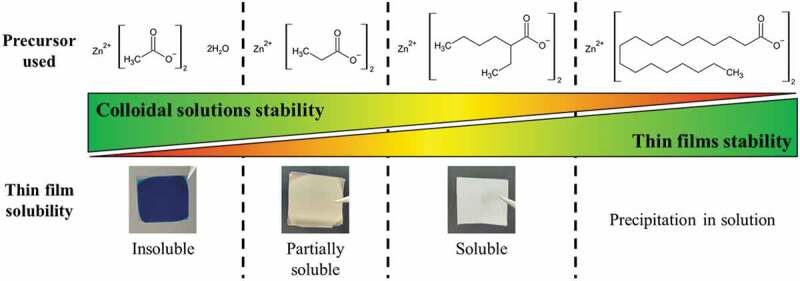
Relation between the zinc-based surface modifier precursors used to prepare the ZnO NCs, the stability of the colloids in solution and the solubility of the obtained thin films by spin-coating.

The NC colloids are prepared following the protocol described by Grasset *et* *al*. [18]. At the end of the synthesis, except for the zinc stearate precursor, the solutions are fully clear and a strong green-yellow emission is observed under UV excitation at 365 nm (Figure 2(a)). These similar optical behaviors clearly indicate comparable NCs’ size (Figure S3), which is expected regarding the robustness of the chemical synthesis protocol used. Indeed, it is admitted that this fast and simple synthesis method yields ZnO NCs, which have a narrow size below 10 nm [19–22]. As example, high-resolution transmission electron microscopy analysis (Figure 2(b)) shows that the NCs functionalized with 2-ethylhexanoate ligands are homogeneous in size with an average diameter of 4.4 nm (±1.2 nm). These solutions can be deposited by spin-coating, producing optical quality thin films (Figure 3). The thickness of the films can be easily adjusted between 60 and 180 nm by changing the deposition conditions (Figure S2).

The proper functionality of the resin for the photostructuring application requires photoinduced crosslinking but also stability of the deposited film to ensure solubility in the non-insolated areas. The chosen ligand must not only ensure repulsion between the particles to promote their redispersibility in the absence of irradiation but also be desorbable and degradable under DUV irradiation to generate a solubility contrast. As shown in Figure 3, this point is very strongly dependent on the nature of the ligands present at the NCs’ surface. This figure shows the four ligands (including the acetate one) considered in this study. In the case of acetate, the colloidal solutions are very stable; however, in a thin film, a coalescence of the NCs is observed when they are exposed to air, which prevents the redissolution of the thin film following its deposition.

Under such conditions, it is not possible to use this primary solution for photostructuring. The increase in the size of the ligands at the NCs surface changes the behavior of the solutions: from propionate to 2-ethylhexanoate, the aggregation of the NCs in the form of a thin film decreases and the film gains in solubility. On the other hand, the stability in time of the solutions decreases and the particles tend to sediment. In the case of stearic precursor, the solution precipitates during the colloid maturation step and is therefore not usable.

In colloidal chemistry, the solvent also plays a critical role in the stability of the colloids but during spin-coating, this solvent is eliminated and only the NCs and the ligands ensuring the repulsion between them remain. Lee *et* *al*. [23] have shown the permeability of the ligand layer of sol-gel synthesized ZnO NCs and this, combined with the small size of the acetate ligands, does not allow sufficient repulsion in the absence of solvent molecules between the particles. As the carbon chain of the ligand increases in size, steric effects are added to counteract the attractive van der Waals forces. Nevertheless, these same longer ligands are less polar and have less affinity for the alcoholic solvent in the solution, which explains the phase separation.

**Figure 4.**
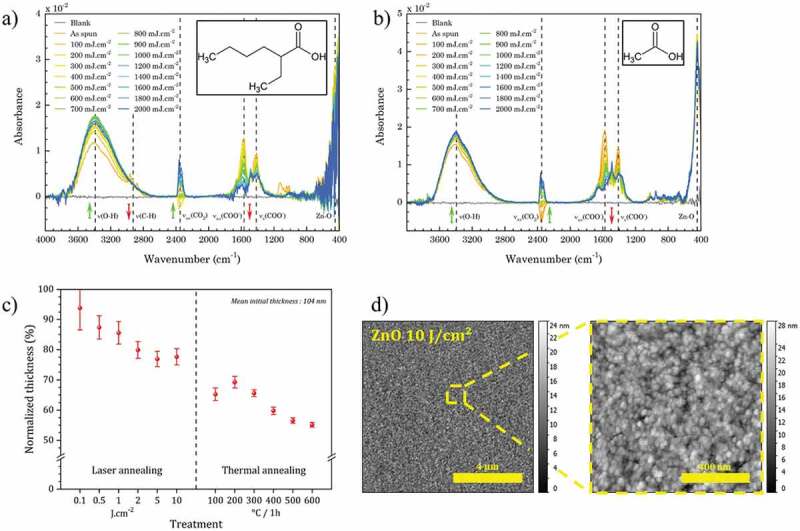
(a) FTIR spectra of ZnO NCs thin film prepared from ZnO:2-ethylhexanoate precursor and their evolution under DUV irradiation. (b) FTIR spectra of ZnO NCs thin film prepared from Zn:2-ethylhexanoate precursor at different ageing time at ambient atmosphere. (c) Evolution of thickness (evaluated by spectroscopic ellipsometry) under DUV and thermal annealing. (d) AFM images of the surface after DUV annealing (10 J.cm^−2^).

The DUV irradiation study was investigated by FTIR spectroscopy in the case of 2-ethylhexanoate ligands. The results are presented in Figure 4.

After spin-coating, we distinguish (Figure 4(a)) the main contributions at 447 cm^−1^ which correspond to ZnO NCs [24] and at 2872 cm^−1^ et 2967 cm^−1^ (νC-H), 1612 cm^−1^ (ν_as_COO^−^) et 1415 cm^−1^ (ν_s_COO^−^) [25,26] which correspond to the ligands on the surface of ZnO NCs. A broad band around 3430 cm^−1^ is attributed to OH functions. In the absence of DUV irradiation, the chemistry of the films remains stable over time (Figure 4(b)).  The absence of a band around 1700 cm^−1^ corresponding to a C=O stretching indicates that the electron in the carboxylate are delocalized. The acidic form isn't detected. Therefore, it implies the absence of uncomplexed ligands in the film.

The DUV irradiation leads to a significant decrease of the ligand bands, which reflects a DUV-induced photolysis of these ligands. Concomitantly, the band at 3430 cm^−1^ increases, which shows the increase of OH. This can be interpreted as oxidation products of ligands and hydroxyls on the surface of ZnO NCs. The non-appearance of the new bands suggests that the species resulting from the photodegradation of the carboxylate ligands are volatile enough to diffuse out of the film.

This degradation can be explained by two possible mechanisms under our conditions. The first one is based on the direct absorption of DUV photons by the ligands which leads to the cleavage of the bonds with energy lower than the incident photon (6.3 eV). A second proposed mechanism involves a photodecarboxylation reaction (also called photo-Kolbe) catalyzed by the ZnO [27,28].

With the disappearance of the ligands, we observe a decrease in the thickness of the deposited film (Figure 4(c)). For a treatment with a dose of 10 J.cm^-2^, the decrease in thickness is about 20%. This value is considerably less important than the shrinkage usually observed when sol–gel solutions are used [29,30]. This shrinkage is also lower than that observed for thermal treatments. This result is related to the use of ZnO NCs that are already pre-crystallized, so the shrinkage can be attributed to the removal of the solvent and ligands as shown in FTIR. Finally, an AFM observation (Figure 4(d)) shows that, after DUV irradiation, the RMS roughness of the 2.7 nm films is of the same order of magnitude as the nanocrystal size.

**Figure 5.**
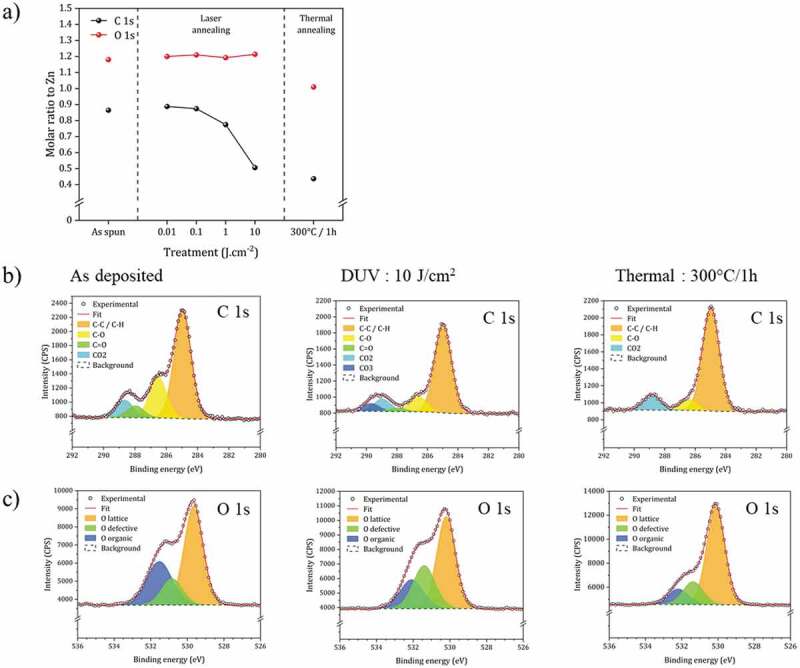
XPS analysis of the material prepared by spin-coating, DUV and thermal annealing. (a) C and O molar ratio (versus Zn) extracted from XPS data (details of data are shown in **Table S1** and **S2**). (b) Analysis of C1s peak. (c) Analysis of O1s peak.

The analysis of the films after DUV treatment was also conducted by XPS. For comparison, thermal treatments were also carried on (300°C/1 h) in order to compare the effect of DUV treatment with such a thermal annealing. The results are shown in Figure 5.

In Figure 5(a) the data are analyzed focusing on the proportions of O and C, relative to Zn. The laser treatment does not affect the O/Zn molar ratio. On the other hand, the C/Zn ratio decreases significantly. These values are compared with the values obtained after heat treatment at 300°C. The O/Zn ratio is slightly lower in the case of the heat treatment and of the same order of magnitude for the C/Zn ratio, demonstrating that both processes are effective in removing the organic part. The efficiency of the heat treatment is slightly more effective.

The fine structure analysis of the C1s and O1s peaks has been conducted. As expected, after spin-coating, contributions that can be extracted from the XPS C1s spectrum (Figure 5(b)) correspond to C-C/C-H, C-O, C=O and CO_2_ and O1s spectrum (Figure 5(c)) correspond to O lattice, defects and organic moieties. DUV irradiation (10 J.cm^-2^) induces a decrease of all these contributions, and especially the C=O, which confirms the photolysis of acetate functions. A carbonate contribution appears which is in agreement with the decarboxylation and oxidation mechanism. CO_2_ formed by ligand degradation may be trapped in thin films by complexation on metals. In samples prepared by thermal treatment, this contribution is not observed, which shows a specific mechanism under DUV irradiation.

For the O1s peak, DUV irradiation causes a significant decrease in the contribution of organic O, which is consistent with the FTIR results. The comparison with the thermal treatment shows that the proportion of O defects remains of the same order of magnitude as for the spin-coated material and much higher than for the heat-treated material. We will see later the impact that this property has on the photoluminescence properties.

**Figure 6.**
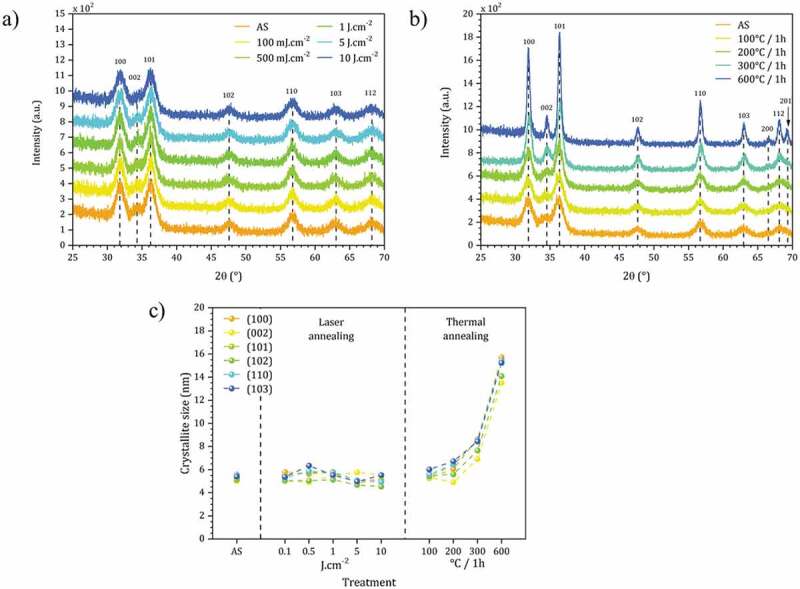
Grazing incidence X-ray diffraction analysis of the ZnO NCs thin films with (a) DUV treatment and (b) thermal treatment. (c) Crystallite size calculated from XRD data using the Scherrer equation for as-spin film (AS) and films after DUV and thermal annealing.

The impact of the DUV process on the nanocrystals has also been evaluated by GIXRD on non-modified ZnO NCs (*i.e.* with acetate as main surface ligands). The objective is to describe the crystal structure and the size of the crystallites in the as-spin-coated film (AS) as well as the evolution of this size during the DUV irradiations compared to thermal treatments. The results are presented in Figure 6.

The diffraction peaks for ZnO are indeed already present on the diffractograms for the AS films as expected. This result confirms that our approach can prepare dense nanocrystalline thin films by simple one-step spin-coating of colloidal nanocrystals, without annealing step.

A qualitative evaluation of the size (D) of the crystallites (in the direction perpendicular to the lattice planes) in the thin film can be done in first approximation by using the Scherrer formula [31]:

$$D_{hkl}=\frac{K\lambda }{\beta_{hkl}\cos\theta }$$

Where

K: form factor (taken as 0.9 rad) [value generally between 0.89 and 0.94],

λ: wavelength of the incident X-ray beam (Kα_1_ line of copper 1.541 Å),

β: width at half-height of the diffraction peak (in rad),

θ: angular position of the diffraction peak (in °),

hkl: the Miller indices of the planes being analyzed.

Directly after spin-coating, the application of the Scherrer formula (at each single diffraction peak) gives an average value D = 5.7 nm (±0.3 nm) [32]. Regarding the anisotropic structure of ZnO and the non-correction of the instrumental contribution for β, this value is an approaching value and only qualitative, nevertheless which is consistent with the value determined by TEM (Figure 2(c)). After DUV irradiations between 0.1 and 10 J.cm^-2^, no significant changes (position, width, relative intensity of diffraction peaks) are observed (Figure 6(a)). We conclude that the morphology of ZnO NCs is stable under these conditions. On the other hand, as expected, by heat treatment in air (at temperatures between 100°C and 600°C), the diffraction lines become sharper as the annealing temperature increases, showing that the conditions for the sintering of ZnO NCs are reached (Figure 6(b)). The average size of the crystallites evolves from 6.0 nm (100°C) to 16.0 nm (600°C) (±0.3 nm). The data are summarized in Figure 6(c).

It is finally remarkable to note that ZnO NCs are very stable under irradiation conditions, which are used to crosslink the material and mineralize it by degradation of the ligands. The central point of this result lies in the possibility of treating the material while remaining at room temperature, since we note that any increase in temperature causes a reorganization of the internal structure of the ZnO NCs (XPS) and also of their size by maturation. This proves once again that the DUV treatment conditions preserve the integrity of the NCs, which is interesting to preserve their photoluminescence properties.

**Figure 7.**
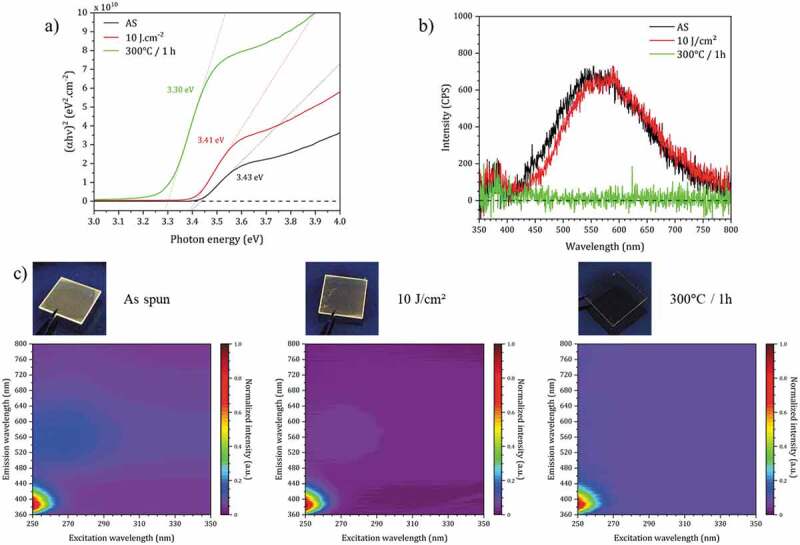
(a) Tauc plots of ZnO NCs thin film (as deposited (AS), with DUV treatment and thermal annealing). (b) Emission spectra of ZnO NCs thin films with different curing conditions under 350 nm excitation. (c) Excitation-emission maps of ZnO NCs thin films with different curing conditions and associated visible luminescence images of the thin film under 365 nm excitation.

These optical properties of the thin films (absorption, photoluminescence) are shown in Figure 7.

Figure 7(a) shows how the band gap of the film evolves for DUV and thermal treatment, and in comparison, with measurements made on the just spin-coated film. By applying the Tauc plot model [33] from absorbance spectra of thin films deposited on quartz substrates (Figure S4), one can evaluate the value of the optical band gap in the three cases. The passage of the absorbance to the representation in Tauc diagram is done thanks to the following formulas:

$${\left(\alpha h\nu \right)}^{n}=K\left(h\nu -E_{g}\right)$$

$$\alpha =\frac{A\cdot ln\left(10\right)}{d}$$

Where α, K, h, ν, d, A, and $E_{g}$ are, respectively, the absorptivity (in nm^−1^), a constant of proportionality (in eV^n-^^1^·nm^n^, depending on the value of the power n), Planck’s constant (equal to 6.62607015 × 10^−34^ J·s), frequency of photons (in Hz), the film thickness (expressed in cm), the absorbance (dimensionless) and the energy of the band gap (in eV). n is a factor depending on the type of band-gap transition allowed or forbidden, direct or indirect, in the case of ZnO n = 2 (direct transition allowed)

The results are very close for the spin-coated and DUV treated films with a band gap value close to 3.42 eV, which is consistent with the estimated size by HRTEM. In the case of the heat-treated film, this value is 3.30 eV, more in agreement with the expected value for bulk ZnO [34].

ZnO is a wide-band gap semiconductor material. Its photoluminescence properties have been widely studied [35–38]. The photoluminescence of ZnO contains two components of emission: an ultraviolet (UV) emission induced by the band gap transition related to a direct recombination of photogenerated charge carriers and a wide emission in the visible range, usually relatively broad, ranging from green to red. This last very broad visible emission is related to the concentration of point, surface and structural defects in the material (doubly ionized zinc vacancies and an ionized interstitial zinc, donor – acceptor pair recombination involving an impurity acceptor, interstitial oxygen and/or oxygen vacancies Vo [39–43]). Van Dijken *et al*. suggested for the appearance of the visible green-yellow band around 2.3 eV the necessity of formation of doubly charged oxygen vacancies. However, the correlation between the role of defects and the broadening observed in this visible emission is not yet fully understood.

Figure 7(b) shows the luminescence spectra of ZnO thin films prepared by our approach, after spin-coating, DUV treatment and thermal annealing, for excitation at 365 nm. In the first two cases, the green-yellow photoluminescence emission is observed. The maxima of the spectra are almost superimposed, which confirms once again that the DUV treatment preserves the integrity of the ZnO NCs. On the other hand, as expected, the luminescence in the visible range is not present for the sample treated at 300°C (*i.e.* the elimination of oxygen vacancy defect and chemisorbed molecules by thermal annealing in air deactivates the visible band), confirming the different impact of the DUV and thermal treatment on this material. Figure 7(c) shows the excitation and emission maps for the 3 treatment conditions. It clearly shows the UV emissions that are observed for all three types of treatment. On the other hand, the visible luminescence is absent for the sample that was heated at 300°C. The photographs showing the samples under UV excitation are consistent with these measurements. We can also verify the transparency of the films deposited on glass (even after annealing at 300°C).

**Figure 8.**
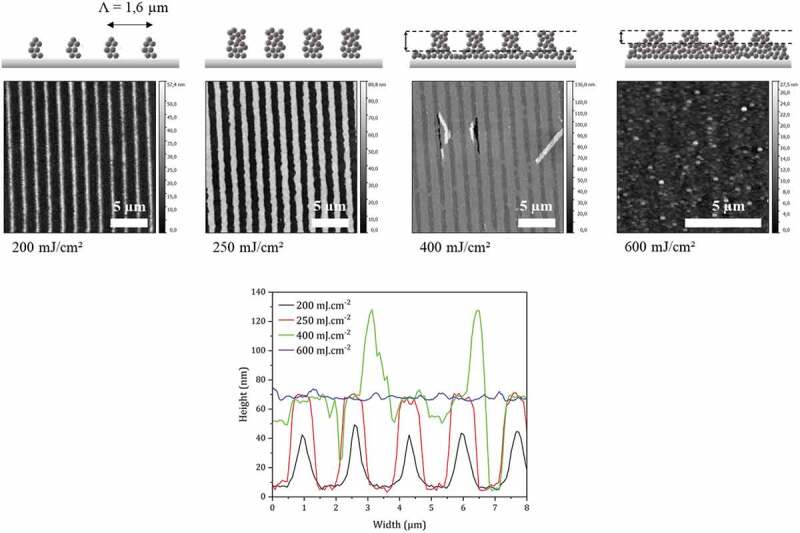
Topography AFM images of photostructured films with a binary mask of 1.6 µm period (line/space ratio :1/1) for doses between 200 and 600 mJ.cm^-2^. Films of 80 nm initial thicknesses are irradiated directly after spin-coating and developed in cyclohexanone for 60 s. Bottom: cross section along x direction for the above four AFM images.

In this last part, we demonstrate and study DUV photopatterning of ZnO NC films (NCs prepared from Zn:2-ethylhexanoate precursor). The goal is to use the DUV-induced ligand photolysis mechanism to create interactions between ZnO NCs and thus change the solubility in the irradiated areas. Figure 8 shows examples of patterns obtained with a binary mask of period 1.6 µm. The doses used vary between 200 mJ.cm^-2^ and 600 mJ.cm^-2^. These doses are therefore much lower than the maxima doses for which the impact of DUV irradiation has been evaluated. In other words, the properties of ZnO NCs are preserved for such values.

For the lowest doses (200 and 250 mJ.cm^-2^), very well-defined patterns are obtained with the silicon wafer uncovered between two lines. The optimal dose is 250 mJ.cm^-2^. For this value, the line/space ratio is 1 and the height of the patterns corresponds to the nominal thickness of the film, corrected for the shrinkage of about 10% that is expected for such conditions. For higher doses (400 and 600 mJ.cm^-2^), the sample is over-irradiated and material is visible between the patterns. Finally, at 600 mJ.cm^-2^, the patterns are no longer visible.

**Figure 9.**
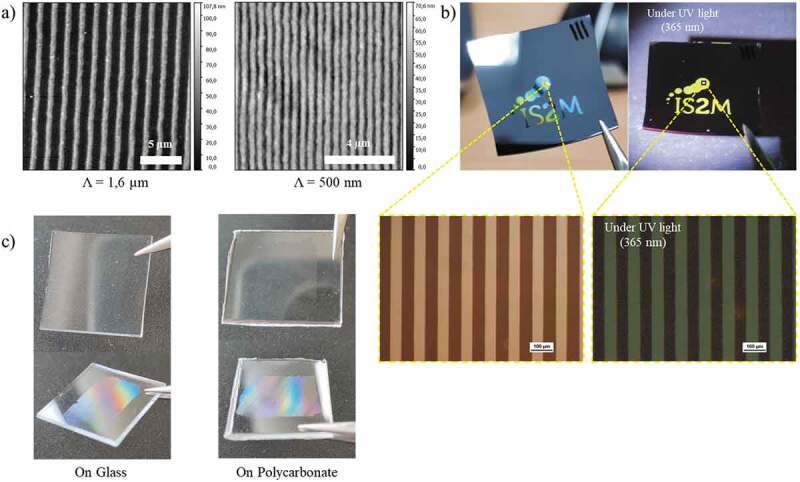
(a) AFM images of 10 µm and 500 nm period microstructures on silicon. (b) Photographs of a sample prepared with a mask showing the laboratory logo under visible and UV illumination and microscopy images under visible and UV illumination (scale bar 100 µm). (c) Examples of ZnO NC arrays prepared on glass and polycarbonate substrates.

This developed approach is very versatile, as demonstrated by the examples shown in Figure 9. The period can be adjusted in a wide range, up to sub-micrometer resolutions (Figure 9(a)). As already demonstrated previously, the DUV process allows to keep the photoluminescence properties and thus to obtain luminescent micro- or nanostructures directly after photopatterning (Figure 9(b)). The other advantage of avoiding any thermal treatment is to make the process compatible with various substrates including silicon, glass and also plastic substrates. An example is shown with a polycarbonate substrate (Figure 9(c)). The low dose required to crosslink the ZnO NCs has no effect on the substrate. Note that the ZnO NC film also acts as a filter that protects the plastic from DUV irradiation.

## 4. Conclusion

The aim of this paper was to demonstrate a new patterning route to integrate ZnO NCs by direct laser writing while maintaining their photoluminescence properties and achieving submicrometer resolutions. These objectives were achieved by combining syntheses of ZnO NCs with suitable surface functionalization to ensure a difference in solubility before and after DUV irradiation (λ = 193 nm). Different zinc-based surface modifiers were tested and the zinc 2-ethylhexanoate demonstrated the optimal properties. On a molecular level, this behavior is achieved by a photoinduced decarboxylation reaction of the ligands that causes the aggregation of the NCs, resulting in their insolubility in the irradiated parts. Under these conditions, colloidal solutions of crystallized ZnO can be used as negative photoresists. The room-temperature process, which does not require thermal post-treatment or etching, therefore appears particularly interesting for integrating these luminescent NCs into complex devices. The process is compatible with plastic substrates, which opens the door to optoelectronic applications on flexible substrates. Ongoing work shows that this work can be extended to other NCs of different natures, which further illustrates the interest of this method.

## Supplementary Material

Supplemental Material
