# Supplementary material for: Deep-UV laser direct writing of photoluminescent ZnO submicron patterns: an example of nanoarchitectonics concept
Source: Sci Technol Adv Mater. 2022 Oct 7;23(1):535–46. doi: 10.1080/14686996.2022.2116294 (PMC9553187; doi:10.1080/14686996.2022.2116294)
Supplement: Supplemental Material [file TSTA_A_2116294_SM0767.pdf]

Supporting Information

Deep-UV Laser Direct Write of Photoluminescent ZnO Sub-micropatterns: An Example of Nanoarchitectonic Concept

Quentin Kirscher[a,b], Samar Hajjar-Garreau[a,b], Fabien Grasset[c,d]*, Dominique Berling[a,b]* and Olivier Soppera[a,b]*

[a]*Institut de Science des Matériaux de Mulhouse (IS2M) UMR 7361 CNRS-UHA, Université de Haute Alsace, Mulhouse, France;*

[b]*Université de Strasbourg, Strasbourg, France;*

[c]CNRS-Saint Gobain-NIMS, IRL 3629, Laboratory for Innovative Key Materials and Structures (LINK), National Institute for Materials Science (NIMS), Tsukuba 305-0044, Japan;

[d]*Univ. Rennes, CNRS, ISCR UMR 6226, ScanMAT UAR 2025, F-35000 Rennes, France.*

E-mail addresses: fabien.grasset@univ-rennes1.fr (F. Grasset); dominique.berling@uha.fr (D. Berling); olivier.soppera@uha.fr (O. Soppera)

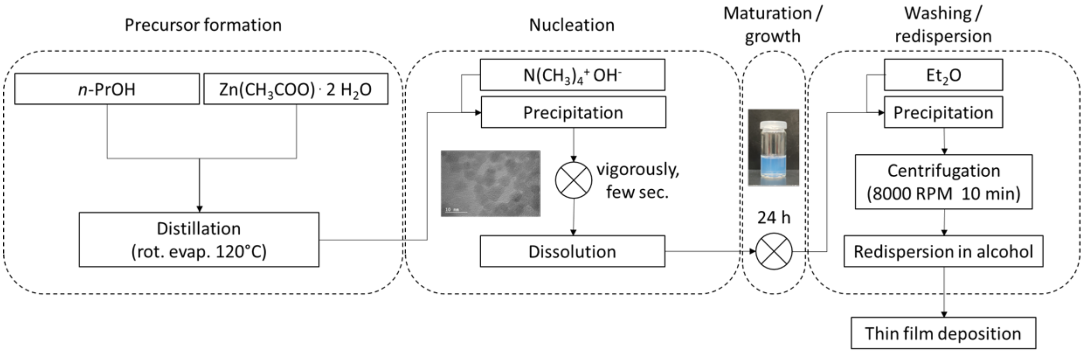

Figure S1 : Schematic view of the protocol for the preparation of the ZnO NP in the case of the acetate precursor

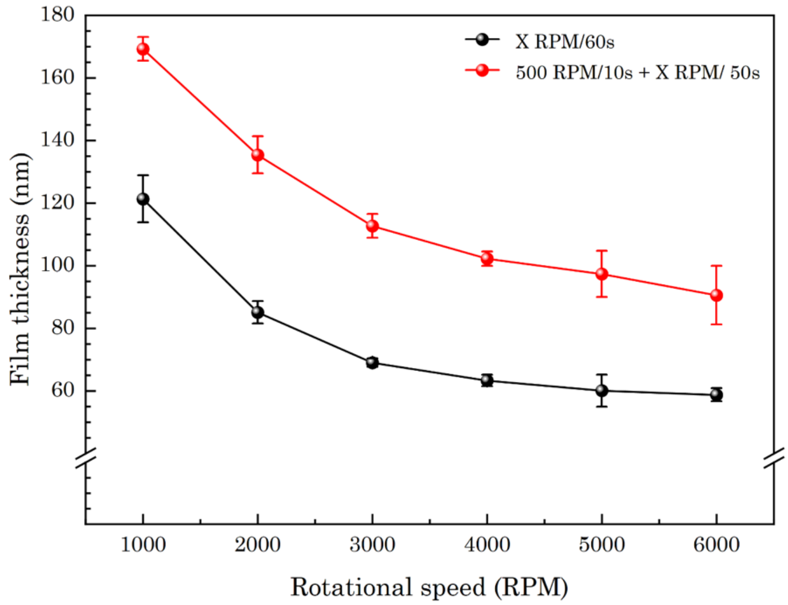

Figure S2: Thickness of the ZnO NP films prepared by spin-coating using different rotation speed. Thickness was determined by spectroscopic ellipsometry.

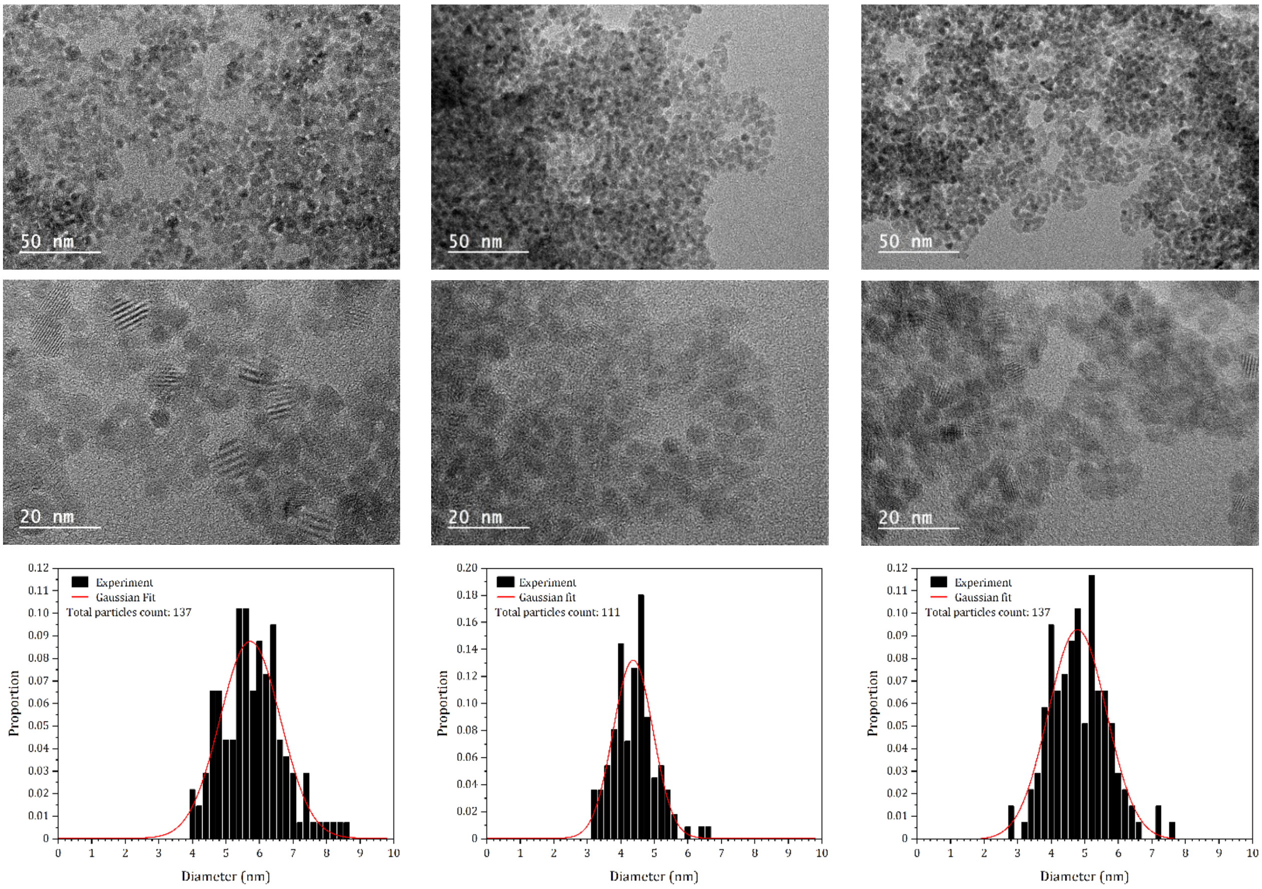

Figure S3: TEM pictures at different magnification of the ZnO NCs functionalized with different carboxylate ligands and associated particle size distribution. From left to right: acetate-capped, 2-ethylxanoate-capped and stearate-capped ZnO NPs

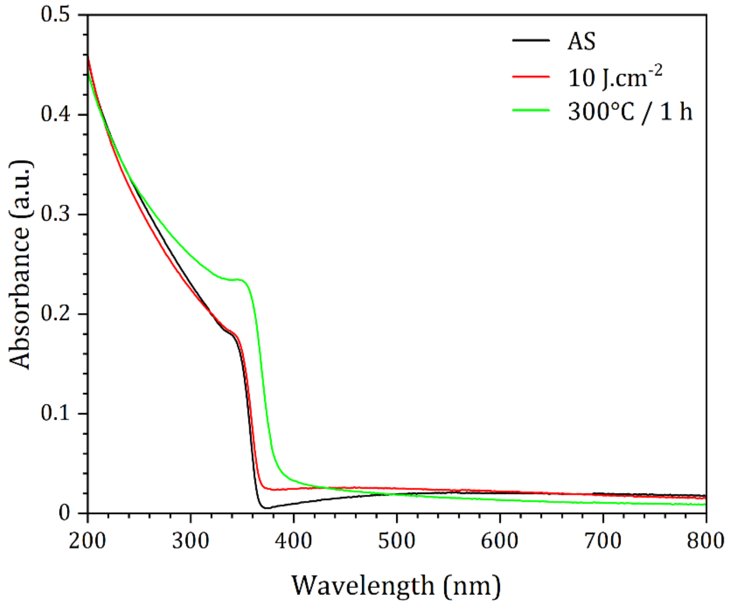

Figure S4: Absorbance spectra of ZnO NCs thin film with different treatment

Table S1: XPS data for ZnO NP thin film treated by DUV.

Sample	C 1s				O 1s				Zn 2p			
	Function	Position (eV)	Area	Proportion (%)	Function	Position (eV)	Area	Proportion (%)	Function	Position (eV)	Area	Proportion (%)
As spun	C-H	284,96	53,11	60,49	O^{2-}	529,67	64,43	53,16	Zn^{2+}	1020,86	101,30	100
	C-O	286,52	20,09	22,88	Defect O	530,84	18,56	15,31				
	C=O	287,97	5,91	6,73	CO	531,54	38,20	31,52				
	COO	288,66	8,69	9,90								
	CO_3	-	-	-								
10 mJ/cm²	C-H	284,94	59,22	64,19	O^{2-}	529,61	46,54	50,34	Zn^{2+}	1020,88	98,55	100
	C-O	286,53	18,65	20,21	Defects	530,71	16,38	17,72				
	C=O	287,93	4,60	4,99	CO	531,49	29,53	31,94				
	COO	288,63	9,79	10,61								
	CO_3	-	-	-								
100 mJ/cm²	C-H	284,97	58,36	64,28	O^{2-}	529,68	59,67	51,10	Zn^{2+}	1020,90	96,33	100
	C-O	286,56	18,94	20,86	Defect O	530,82	21,35	18,28				
	C=O	287,96	5,28	5,82	CO	531,57	35,75	30,62				
	COO	288,66	8,21	9,04								
	CO_3	-	-	-								
500 mJ/cm²	C-H	284,96	44,04	61,71	O^{2-}	529,63	40,41	43,18	Zn^{2+}	1020,70	74,15	100
	C-O	286,53	17,12	23,99	Defect O	530,13	9,49	10,14				
	C=O	287,83	4,14	5,80	CO	531,23	43,69	46,68				
	COO	288,60	6,07	8,50								
	CO_3	-	-	-								
1 J/cm²	C-H	284,94	47,14	61,58	O^{2-}	529,73	59,84	49,92	Zn^{2+}	1020,92	93,41	100
	C-O	286,52	19,59	25,59	Defect O	530,95	30,85	25,74				
	C=O	287,94	4,69	6,13	CO	531,69	29,17	24,34				
	COO	288,80	5,13	6,70								
	CO_3	-	-	-								
5 J/cm²	C-H	284,96	38,40	66,17	O^{2-}	530,06	55,32	51,88	Zn^{2+}	1021,05	87,17	100
	C-O	286,61	9,55	16,46	Defect O	531,03	11,37	10,66				
	C=O	-	-	-	CO	531,63	39,94	37,46				
	COO	288,40	6,22	10,72								
	CO_3	289,15	3,86	6,65								
10 J/cm²	C-H	248,97	38,96	69,16	O^{2-}	530,22	72,91	51,52	Zn^{2+}	1021,38	114,76	100
	C-O	286,60	6,83	12,12	Defect O	531,40	39,99	28,26				
	C=O	287,97	1,59	2,82	CO	532,09	28,63	20,23				
	COO	288,97	5,50	9,76								
	CO_3	289,70	3,45	6,12								

Table S2 : XPS data for ZnO NP thin film treated by thermal annealing.

Sample	C 1s				O 1s				Zn 2p			
	Function	Position (eV)	Area	Proportion (%)	Function	Position (eV)	Area	Proportion (%)	Function	Position (eV)	Area	Proportion (%)
100°C / 1 h	C-H	284,96	43,83	61,71	O^{2-}	529,64	48,12	52,29	Zn^{2+}	1020,66	75,19	100
	C-O	286,42	16,55	23,30	Defect O	530,45	0,52	0,57				
	C=O	287,54	4,46	6,28	CO	531,07	43,39	47,15				
	COO	288,55	6,19	8,71								
	CO_3	-	-	-								
200°C / 1 h	C-H	284,98	22,18	78,35	O^{2-}	530,22	30,71	58,44	Zn^{2+}	1021,21	46,41	100
	C-O	286,52	2,08	7,35	Defect O	531,18	10,17	19,35				
	C=O	-	-	-	CO	532,04	11,67	22,21				
	COO	288,82	4,05	14,31								
	CO_3	-	-	-								
300°C / 1 h	C-H	284,97	42,61	79,33	O^{2-}	530,13	94,12	69,86	Zn^{2+}	1021,27	138,26	100
	C-O	286,44	4,61	8,58	Defect O	531,38	23,90	17,74				
	C=O	-	-	-	CO	532,20	16,71	12,40				
	COO	288,83	6,49	12,08								
	CO_3	-	-	-								
600°C / 1 h	C-H	284,96	16,89	80,81	O^{2-}	530,22	39,03	70,43	Zn^{2+}	1021,16	52,84	100
	C-O	286,63	1,60	7,66	Defect O	531,28	8,89	16,04				
	C=O	-	-	-	CO	532,16	7,50	13,53				
	COO	288,72	2,41	11,53								
	CO_3	-	-	-								
